# Does liver resection/transplantation affect respiratory induced liver motion in patients with hepatocellular carcinoma?

**DOI:** 10.1002/acm2.12113

**Published:** 2017-06-06

**Authors:** Yong Hu, Yong‐Kang Zhou, Yi‐Xing Chen, Lu‐Xi Ye, Zhao‐Chong Zeng

**Affiliations:** ^1^ Department of Radiation Oncology Zhongshan Hospital Fudan University Shanghai China

**Keywords:** four‐dimensional computed tomography, liver motion, liver resection, liver transplantation

## Abstract

The purpose of this study was to evaluate the changes in magnitude of three‐dimensional (3D) liver motion after liver resection/transplantation in patients with hepatocellular carcinoma (HCC) using four‐dimensional (4D)‐computed tomography (CT) images. From January 2012 to April 2016, 74 HCC patients underwent 4D‐CT scans under a free‐breathing state to assess respiratory liver motion. Of the 74 patients, 40 did not have a liver resection/transplantation (Group A), 34 with liver resection/transplantation. 15 underwent major or minor resection in the right liver lobe (Group B), 14 underwent major or minor resection in the left liver lobe (Group C), and five underwent liver transplantation (Group D). The 4D‐CT images were sorted into 10 image series according to the respiratory phase from the end inspiration to the end expiration, and then transferred to treatment planning software. All liver contours were drawn by a single physician and confirmed by a second. Liver relative coordinates were automatically generated to calculate liver respiratory motion in different axial directions and compiled into a single composite image. Differences in respiratory liver motion were assessed using one‐way ANOVA. The average liver respiratory motion in the cranial‐caudal direction and 3D magnitude were 10.46 ± 2.78 mm (range, 5.60–18.80 mm) and 11.74 ± 2.65 mm (range, 7.45–20.79 mm) for patients without liver resection/transplantation, and 7.74 ± 2.79 mm (range, 2.20–12.90 mm) and 9.07 ± 2.38 mm (range, 4.79–14.08 mm) for posthepatectomy/post‐transplant patients respectively. There were significant differences between Group A and B, Group A and C, Group A and D. However, there were no significant differences among Group B, C, and D. Liver resection/transplantation greatly affected respiratory‐induced liver motion in patients with HCC. We, therefore, recommend discriminatory internal target volume (ITV) determination for patients with or without liver resection/transplantation undergoing external radiotherapy for hepatic tumors while respiratory motion management is unavailable.

## 1 INTRODUCTION

Hepatocellular carcinoma (HCC) is a highly prevalent and lethal neoplasia,^1^ comprising the majority of primary liver cancers worldwide (70–90%). An estimated 782,500 new liver cancer cases and 745,500 deaths occurred worldwide in 2012 due to HCC, with China alone accounting for approximately 50% of the total number of cases and deaths.^2^ The preferred treatments for HCC are surgical resection and percutaneous destruction methods (uni‐ and multipolar radiofrequency, microwave, cryotherapy, and electroporation). In selected patients, liver transplantation is the best treatment option for small HCC with severe liver cirrhosis.^3^ Curative therapies (resection, transplantation, and ablation) can improve survival in patients diagnosed at an early stage of HCC and offer a potential long‐term cure.^1^, ^4^ However, metastasis is the major risk factor of HCC, which impacts long‐term survival of patients with posthepatectomy HCC, and contributes to the high recurrence rate.^5^, ^6^ Post‐transplant HCC recurrence is reported in up to 25% of cases and drastically affects patient survival.^7^, ^8^, ^9^, ^10^ External beam radiotherapy (EBRT) is widely used for HCC in Asia,^11^ and when used in combination with hepatic arterial embolization, is a promising treatment.^12^ In addition, with current advancements in precision radiotherapy, stereotactic body radiation therapy (SBRT) has also become a promising alternative treatment for patients with primary or recurrent small HCC who are considered unsuitable for surgical resection or local ablative therapy.^13^, ^14^ EBRT may play an important role in preventing post‐transplant or postoperative recurrence of and/or metastasis from HCC.^13^, ^15^, ^16^, ^17^, ^18^

Patients with unresectable but limited HCC recurrence may undergo EBRT, but the hepatic tumors move during EBRT due to respiratory‐induced liver motion. In order to avoid both inadequate tumor coverage and unnecessary liver parenchyma irradiation, it is crucial to determine the internal target volume (ITV). The ITV boundary range primarily relies upon respiration‐induced liver motion, and if not properly accounted for, motion of this magnitude could lead to altered dosimetry due to use of a static plan and irradiation of an uncertain volume of normal tissue.^19^, ^20^ Inaccurate definitions of the volume of a hepatic tumor and normal tissue could lead to a greater risk of toxicity. Although there are benefits to defining individual ITV, the data are obtained using four‐dimensional computed tomography (4D‐CT), but the process of contouring each phase is time‐consuming and labor‐intensive. The gross target volume (GTV) must be manually contoured to form ITV in all respiratory phases of a 4D scan image. In addition, the 4D‐CT technique is not universally available in all radiation oncology centers, and some radiation oncologists may determine the margin ITV based upon their individual experience. In theory, ligament damage and tissue adhesions surrounding a remnant liver may cause a decrease of amplitude in respiratory‐induced liver motion. To date, the impact on ITV margins after liver resection in HCC patients has not been reported. Therefore, in this study, we investigated the differences in liver motion between post‐transplant or postoperative recurrence HCC patients and unresectable HCC patients in a free‐breathing state to provide a valuable reference for radiation oncologists when determining ITV.

## 2 MATERIALS AND METHODS

### 2.A Patients

Patient inclusion criteria were: (a) confirmed HCC and plan to receive EBRT; (b) presence of hepatic tumors; (c) Child‐Pugh A liver function and Karnofsky performance status >80; (e) no colostomy or ascites; (f) no history of chest surgery; (g) regular breathing after basic breath training; and (h) no disease affecting pulmonary function.

Patient demographics and clinical characteristics are shown in Table 1. Between January 2012 and April 2016, 74 consecutive patients (59 male and 15 female; age range 22–84 yr) diagnosed with HCC were divided into four groups (described in more detail below) and underwent 4D‐CT scans to assess respiratory liver motion.

**Table 1:** Patient demographics and clinical characteristics

	Group A (*n* = 40)	Group B (*n* = 15)	Group C (*n* = 14)	Group D (*n* = 5)	*P*‐value
Gender					0.516
Male	30 (75.0%)	13 (86.7%)	11 (78.6%)	5 (100.0%)	
Female	10 (25.0%)	2 (13.3%)	3 (21.4%)	0 (0.0%)	
Age (years)					0.114
≤60	21 (52.5%)	7 (46.7%)	10 (71.4%)	5 (100.0%)	
>60	19 (47.5%)	8 (53.3%)	4 (28.6%)	0 (0.0%)	
Height (cm)					0.712
≤170	28 (70.0%)	9 (60.0%)	11 (78.6%)	3 (60.0%)	
>170	12 (30.0%)	6 (40.0%)	3 (21.4%)	2 (40.0%)	
Weight (kg)					0.121
≤70	20 (50.0%)	10 (66.7%)	12 (85.7%)	3 (60.0%)	
>70	20 (50.0%)	5 (33.3%)	2 (14.3%)	2 (40.0%)	
BMI					0.411
<18.5	3 (7.5%)	2 (13.3%)	0 (0.0%)	0 (0.0%)	
18.5 ≤ BMI < 24	14 (35.0%)	8 (53.3%)	9 (64.3%)	3 (60.0%)	
24 ≤ BMI < 28	16 (40.0%)	3 (20.0%)	5 (35.7%)	2 (40.0%)	
28≤BMI	7 (17.5%)	2 (13.3%)	0 (0.0%)	0 (0.0%)	
Tumor location					0.189
Intrahepatic	29 (72.5%)	9 (60.0%)	11 (78.6%)	1 (20.0%)	
Intrahepatic+LNM	4 (10.0%)	3 (20.0%)	2 (14.3%)	1 (20.0%)	
Intrahepatic+distantmetastasis	7 (17.5%)	3 (20.0%)	1 (7.1%)	3 (60.0%)	
Tumor in liver					0.585
Right lobe	26 (65.0%)	13 (86.7%)	12 (85.7%)	4 (80.0%)	
Left lobe	6 (15.0%)	1 (6.7%)	1 (7.1%)	0 (0.0%)	
Left and right lobes	8 (20.0%)	1 (6.7%)	1 (7.1%)	1 (20.0%)	
Intrahepatic lesions					0.561
Solitary	27 (67.5%)	11 (73.3%)	10 (71.4%)	2 (40.0%)	
Multiple nodules	13 (32.5%)	4 (26.7%)	4 (28.6)	3 (60.0%)	
Diameter (cm)					0.574
≤5	26 (65.0%)	11 (73.3%)	12 (85.7%)	5 (100.0%)	
5~10	10 (25.0%)	3 (20.0%)	2 (14.3%)	0 (0.0%)	
≥10	4 (10.0%)	1 (6.7%)	0 (0.0%)	0 (0.0%)	

BMI, body mass index; LNM, lymph node metastasis. Distant metastasis included adrenal gland metastasis and bone metastasis in this study.

### 2.B Grouping methods

Patients were divided into Groups A, B, C, and D as follows: 40 patients with unresectable HCC (Group A), 15 patients who underwent major or minor resection in the right lobe of the liver (Group B), 14 patients who underwent major or minor resection in the left lobe of the liver (Group C), and 5 patients who received liver transplantation (Group D). Each patient underwent basic respiratory training guided by a radiotherapy oncologist and therapist before 4D‐CT image acquisition.

### 2.C 4D‐CT image acquisition

4D‐CT scans were obtained using a Big Bore CT Scanner (Siemens Somatom CT, Sensation Open; Siemens Healthcare, Munchen Germany). Patients were placed in a supine position with arms raised above the forehead, and were immobilized using a vacuum cushion. The X‐ray tube settings were: 120 KV; 400 mAs; Pitch 0.1; Gantry rotation cycle time 0.5 s; 3 mm reconstructed thickness. The respiratory phase on the respiratory wave was manually adjusted and confirmed by the CT‐simulation technician prior to CT image reconstruction. 4D‐CT images from respiratory raw data were sorted into a 10 CT image series (CT0~CT90) according to the respiratory cycle, with CT0 being defined as the end inspiration phase and CT50 as the end expiration phase.^21^ Datasets for 4D‐CT scans were then transferred to Nucletron Oncentra's treatment planning software Version 4.3(NUCLETRON B.V., Veenendaal, Netherlands), and all liver contours were drawn by an experienced observer (HY) and confirmed by a single physician (YKZ).

### 2.D Liver displacement acquisition and analysis

**Figure 1:**
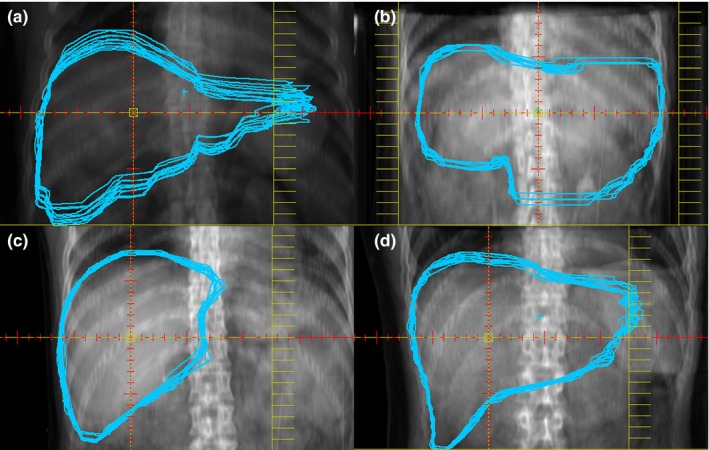
An overlay of 10 liver contours rendered on a digitally reconstructed radiography image showing the different respiratory‐induced liver motions for four groups. The image in (a) is from a Group A patient, the image in (b) is from a Group B patient, the image in (c) is from a Group C patient, and the image in (d) is from a Group D patient.

Liver contours were delineated at all CT image phases and then copied manually to a single plan. Nine liver contours of CT10~CT90 were copied onto the CT0 image, and were designated CopyContour10~CopyContour90. There were 10 liver contours (CopyContour10~CopyContour90 and liver contours of CT0) on the CT0 image. An AP digitally reconstructed radiography image was created to order visualize each phase contour (Fig. 1). The relative coordinates of the liver were automatically generated to calculate the respiratory liver motion in different axial directions. The position for each liver was expressed using the left‐right (LR), cranial‐caudal (CC), and anterior‐posterior (AP) coordinates of the center of mass (COM) for each 4D‐CT bin. Then, the range of respiratory liver motion from the COM of each coordinate was obtained. The maximum range of motion in each axial direction was obtained by subtracting the minimum relative coordinate value from the maximum relative coordinate value. The 3D motion magnitude of the COM was calculated according to the following formula:

$$V={{(}_{\Delta }{\text{LR}}^{2}{+}_{\Delta }{\text{CC}}^{2}{+}_{\Delta }{\text{AP}}^{2})}^{1/2}$$

Variables were expressed as the mean ± standard deviation.

### 2.E Statistical analyses

A Chi‐square (*χ* ^2^) test was used to compare patient demographics and clinical characteristics between the four patient groups (A‐D). The variation between the four groups in the LR, CC, AP, and 3D directions were assessed using a one‐way ANOVA test, using Student's t‐test to compare breath amplitude of patients with and without liver resection/transplantation, and liver motion by different postoperative time nodes (Table 5). Post Hoc Test was used to perform multiple comparisons of liver motions among the four groups (Table 4). The cut‐off for significance was *P *< 0.05. All calculations were performed using SPSS 15.0 for Windows (Chicago, Illinois, USA).

## 3 RESULTS

### 3.A Respiratory liver motion

Table 2 lists the breath amplitude of patients with and without liver resection/transplantation in the relative LR, CC, AP, and 3D axial directions. The average liver respiratory motion in the CC direction and 3D magnitude were 7.74 ± 2.79 mm and 9.07 ± 2.38 mm for patients with liver resection/transplantation, and 10.46 ± 2.78 mm and 11.74 ± 2.65 mm for patients without liver resection/transplantation. As shown in Table 2, there was a significant difference in respiratory liver motion between the two groups, although there was no difference in the relative LR and AP directions between the two groups (*P *> 0.05). Liver amplitudes in CC directions ranged from 2.20 to 12.90 mm for patients with liver resection/transplantation, and from 5.60 to 18.80 mm for patients without liver resection/transplantation.

**Table 2:** Breath amplitude (mm) of 74 patients with and without liver resection/transplantation

Liver resection/transplantation	LR	CC	AP	3D magnitude
Resection/transplantation (*n *= 34)	2.93 ± 1.46	7.74 ± 2.79	2.48 ± 0.98	9.07 ± 2.38
No resection/transplantation (*n* = 40)	3.39 ± 2.08	10.46 ± 2.78	3.08 ± 1.63	11.74 ± 2.65
Maximum (with)	7.30	12.90	4.70	14.08
Maximum (without)	13.20	18.80	8.80	20.79
Minimum (with)	1.10	2.20	1.20	4.79
Minimum (without)	1.20	5.60	0.90	7.45
T	1.071	4.189	1.883	4.520
P	0.288	<0.001	0.064	<0.001

T means T‐value in Student's t test; *P* means *P*‐value in Student's t test**.**

Table 3 lists the respiratory liver motion in the relative LR, CC, AP, and 3D axial directions for each patient group. Respiratory liver motion was anisotropic, and differences were manifested in all axial directions in four respiration states (see Table 3), particularly in the CC direction. The average liver respiratory motion in the CC direction was 10.46 ± 2.78 mm (range 5.60–18.80 mm), 8.11 ± 2.96 mm (range 2.60–12.90 mm), 7.86 ± 2.58 mm (range 3.90–12.70 mm), and 6.26 ± 2.96 mm (range 2.20–8.60 mm) in Groups A, B, C, and D, respectively, with a significant difference in respiratory liver motion among the four groups (*P *< 0.05).

**Table 3:** The magnitude of respiratory liver motion (mm) in different axial directions among the four patient groups

	LR	CC	AP	3D
Group A (*n* = 40)	3.39 ± 2.08	10.46 ± 2.78	3.08 ± 1.63	11.74 ± 2.65
Group B (*n* = 15)	3.44 ± 1.36	8.11 ± 2.96	2.75 ± 0.93	9.81 ± 2.44
Group C (*n* = 14)	2.26 ± 1.18	7.86 ± 2.58	2.41 ± 1.06	8.72 ± 2.36
Group D (*n* = 5)	3.26 ± 1.94	6.26 ± 2.96	1.88 ± 0.69	7.81 ± 1.83
*P*‐value	0.228	0.001	0.176	<0.001

Data are presented as the mean ± standard deviation**.**

Table 4 shows the Multiple comparisons among the four groups using Post Hoc Test. There were significant differences between Group A and B (*P *= 0.007), Group A and C (*P* = 0.004), GroupA and D (*P *= 0.002). However, there were no significant differences among Group B, C, and D (all *P *> 0.05).

**Table 4:** Multiple comparisons of liver motions (mm) in CC and 3D magnitude among the four groups using Post Hoc Test

Axial	(I) Group	(J) Group	Mean difference (I‐J)	Std. error	Sig.	95% Confidence interval	
						Lower bound	Upper bound
CC	A	B	2.35	0.84	0.007	0.66	4.03
		C	2.59	0.87	0.004	0.86	4.32
		D	4.20	1.32	0.002	1.56	6.83
	B	A	−2.35	0.84	0.007	−4.03	−0.66
		C	0.24	1.04	0.816	−1.83	2.31
		D	1.85	1.44	0.204	−1.03	4.72
	C	A	−2.59	0.87	0.004	−4.32	−0.86
		B	−0.24	1.04	0.816	−2.31	1.83
		D	1.60	1.45	0.273	−1.29	4.50
	D	A	−4.20	1.32	0.002	−6.83	−1.56
		B	−1.85	1.44	0.204	−4.72	1.03
		C	−1.60	1.45	0.273	−4.50	1.29
3D	A	B	1.93	0.76	0.014	0.41	3.45
		C	3.01	0.78	0.000	1.45	4.57
		D	3.92	1.19	0.002	1.54	6.30
	B	A	−1.93	0.76	0.014	−3.45	‐0.41
		C	1.08	0.94	0.251	−0.78	2.95
		D	2.00	1.30	0.129	−0.60	4.59
	C	A	−3.01	0.78	0.000	−4.57	−1.45
		B	−1.08	0.94	0.251	−2.95	0.78
		D	0.91	1.31	0.489	−1.70	3.53
	D	A	−3.92	1.19	0.002	−6.30	−1.54
		B	−2.00	1.30	0.129	−4.59	0.60
		C	−0.91	1.31	0.489	−3.53	1.70

The mean difference is significant at the 0.05 level. “Sig.”stands for “*P* value”.

Table 5 lists the respiratory liver motion of patients with liver resection/transplantation over time. At postoperative times of 3, 6, 12, 24, and 36 months for the contrast node, there was no significant difference between the two groups (before and after the postoperative time node) (*P *> 0.05). Respiratory‐induced liver motion would not change much over time after HCC patients received liver resection/transplantation.

**Table 5:** Comparison and analysis of the respiratory liver motion (mm) in 3D magnitude in patients with liver resection/transplantation at different postoperative periods

Postoperative time (months)	N	Mean ± standard deviation (mm)	*P*‐value
≤3	5	7.89 ± 2.23	0.234
>3	29	9.27 ± 2.38	
≤6	8	7.86 ± 2.67	0.100
>6	26	9.44 ± 2.32	
≤12	11	8.53 ± 2.35	0.370
>12	23	9.33 ± 2.40	
≤24	18	8.88 ± 2.47	0.624
>24	16	9.28 ± 2.33	
≤36	22	9.02 ± 2.32	0.866
>36	12	9.16 ± 2.58	

### 3.B Distribution of CC displacement in the four patient groups

**Figure 2:**
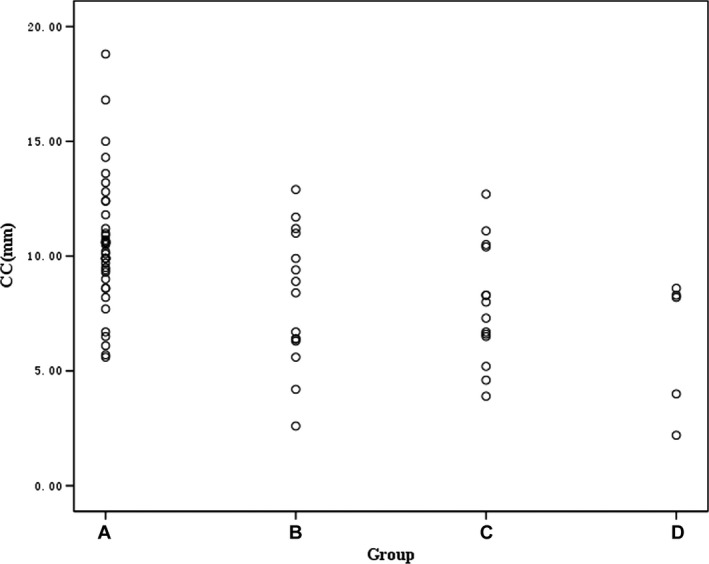
Scatter plot of respiratory liver motion in the CC direction in the four patient groups.

As shown in Fig. 2, the breath amplitude of all HCC patients without liver resection/transplantation in a free‐breathing state did not drop below 5 mm (47.50% of patients reached 5–10 mm, 52.50% of patients reached >10 mm) in the CC direction. Of all HCC patients with a liver resection/transplantation, the breathing amplitude of 17.65% of patients was less than 5 mm, while 23.53% of patients had a liver displacement >10 mm in a free‐breathing state.

## 4 DISCUSSION

The human body achieves gas exchange with its surrounding environment primarily with respiratory motion, using diaphragmatic muscles for breathing. The diaphragm pulls on the liver via a ligament, which induces liver motion, while some ligaments surrounding the liver that are not attached to the diaphragm limit respiratory‐induced liver motion. Thus, ligament function is a critical factor for respiratory‐induced liver motion amplitude. Liver resection and liver transplantation can cause detachment of the ligaments involved in liver respiratory motion. A right‐sided hepatectomy may cause detachment of, among others, the hepatorenal ligament, the round ligament of the liver, the hepatic falciform ligament, the right coronary ligament, and the right triangle ligament. A left‐sided hepatectomy may cause detachment of the round ligament of the liver, the hepatic falciform ligament, the left coronary ligament, the left triangle ligament, and the hepatogastric ligament, among others. During a liver transplant, all peri‐hepatic ligaments will be cut.^22^ Correspondingly, this study found that respiratory‐induced liver motion was smaller in HCC patients with liver resection/transplantation compared to those without liver resection/transplantation.

Some researchers believe that using the COM of liver for analysis overly condenses the data and may not be representative of liver motion. In addition, there is also concern that the reproducibility of manually drawing liver contours impacts accuracy. In this study, we explored respiratory‐induced liver motion primarily from a macro‐perspective. In fact, we did attempt the method of “border locations”. However, quantitative analysis is difficult for two reasons: (a) The drawing error would become bigger using the “border location” method than the error (<0.2 mm) in the COM method, which leads to weak quantitative accuracy; and (b) The inconsistency of liver‐induced “border location” motion may occur. “Border location” motion may not necessarily equal liver motion.^23^ The maximum “border location” motion was very difficult to find, but we still considered “border location” an effective and intuitive method, as illustrated in the representative liver motion images in Fig. 1 from a qualitative perspective. A certain drawing error could inevitably exist in this study, and we explored this issue before we initiated the study. The liver contours were drawn five times in the same patient's 4D‐CT image at different times by a single radiation oncologist (HY), and then the drawing error was compared. The differences of each coordinate value among the COM of five liver contours drawn in the same patient's 4D‐CT image were all less than 0.2 mm, which was deemed acceptable in this study. Therefore, we determined that the liver contours drawn by HY were reproducible, and the drawing error would not impact the accuracy by which COM (and thus motion magnitudes) were determined.

In theory, reduced liver motion can lead to reduced ITV.^24^, ^25^ Therefore, it is important to manage and/or account for respiratory liver motion through means such as abdominal compression (AC),^26^ which uses a constant force applied to the abdomen to reduce liver motion, respiratory gating techniques^27^ to deliver radiation only to the tumor during the respiratory cycle, and active breathing control (ABC),^28^ which achieves temporary and reproducible inhibition of respiration‐induced motion by monitoring the patient's breathing cycle and implementing a breath hold at a predefined stage of respiration and air flow direction. However, each technology has its own indications. For example, patients with risk of thrombosis or colostomy could not undergo AC,^26^, ^29^ and patients with poor breath holding may not undergo ABC; these HCC patients would receive EBRT in a free‐breathing state. Helical tomotherapy is a technique for overcoming the effects of respiration during abdominal tumor radiotherapy.^30^, ^31^ All patients in this study had undergone helical tomotherapy in a free‐breathing state in our institution. However, due to the lack of 4D‐CT equipment in some radiotherapy institutions, radiation oncologists must rely on their own experience to determine the ITV, which is critical for EBRT success in HCC patients with an intrahepatic tumor. Radiation oncologists should consider respiratory‐induced liver motion differently for HCC patients with or without liver resection/transplantation, which is crucial in estimating ITV.

In fact, intrahepatic tumor motion is not equal to respiratory‐induced liver motion.^23^ Technologies explored by radiation oncologists include: (a) Implantation of gold fiducial markers in the healthy liver tissue surrounding the tumor;^32^ however, due to the invasiveness and technical complexity of this technique, it is difficult to popularize in radiation institutions; and (b) Performing contrast‐enhanced (CE) 4D‐CT scans. Although Beddar et al.^33^ developed a tumor‐specific protocol for 4D‐CT imaging of liver tumors using synchronized intravenous (IV) contrast injection to improve the accuracy of tumor delineation for treatment planning, only intrahepatic metastases or cholangiocarcinomas can be successfully imaged in the portal venous phase, a phenomenon that we agreed with. In future studies, we will explore more precise methods to obtain the intrahepatic tumor motion in HCC patients with or without liver resection and transplantation to determine the ITV. Besides, the patient number, especially that in Group B, C, and D, should be expanded to yield more solid conclusion.

Whether liver motion can be used as a tumor motion surrogate in clinical practice is still disputed at present. Kirlivola et al.^34^ reported that the liver tumor motion measured on cine‐MRI did not correlate well with the diaphragm motion measured on fluoroscopy. Balter et al.^23^ demonstrated that the range of ventilatory movement of different locations within the liver could be predicted by diaphragm position to an accuracy that matched or exceeded existing systems for ventilatory tracking. Balter et al.^23^ indicated that liver motion was similar to the diaphragm motion. Recently, Yang et al.^35^ found that liver tumor motion had good correlation with diaphragm motion in the CC and AP directions, and the small magnitude of liver tumor motion in LR direction might be clinically irrelevant. The magnitude of liver motion in CC direction in this study is similar to that reported by Hallman et al.^36^ They also used 4DCT to quantify multiorgan respiration‐induced motion in the abdomen, and found that the average liver motion and liver tumor motion were 7.8 ± 2.6 (range, 3–13) mm and 9.7 ± 5.0 (range, 3–18) mm in CC direction.^36^ Kirilova et al.^34^ reported liver tumor motion on cine‐MRI, the average LR motion was 7.5 mm (range, 3.8–14.8), the CC motion was 15.5 mm (range, 6.9–35.4), and the AP motion was 10 mm (range, 3.7–21.6). In their study, anteroposterior fluoroscopy revealed that the average diaphragm motion in the CC direction was 15 ± 7 mm (range, 5–41 mm).^34^ Akino et al.^25^ reported the maximum ranges of liver tumor motion on cine‐MRI were 2.4 ± 1.4 mm (range, 1.0–5.0 mm), 14.7 ± 5.9 mm (range, 7.4–23.4 mm), and 4.4 ± 3.3 mm (range, 0.8–9.4 mm) in LR, CC, and AP directions respectively. Magnitude of liver motions in this study were smaller than that reported by Kirilova^34^ but similar to that reported by Fernandes,^37^ who used cine‐MRI to measure the liver motion. Kirilova^34^ calculated tumor motion using the maximal tumor edge differences rather than frequency percentiles, which was likely affected by irregularities in breathing amplitude. Fernandes et al.^37^ demonstrated that cine‐MRI detected differences in hepatic tumor motion when compared with 4DCT, cine‐MRI motion was larger than 4DCT for the CC direction in 50% of patients by a median of 3.0 mm (range, 1.5–7 mm), the AP direction in 44% of patients by a median of 2.5 mm (range, 1–5.5 mm), and LR in 63% of patients by a median of 1.1 mm (range, 0.2–4.5 mm). They considered that the cine‐MRI had better time resolution and was better able to capture the extreme positions of the tumor motion than 4DCT as the reason.^37^ More studies are required to investigate the phenomenon and identify which is the real liver tumor motion based on different imaging modalities.

4D‐CT is helpful to determine the internal target volume, but if 4D‐CT is not available then the data of the result in this study could be used along with published margins recipes to determine population‐based target volumes. If possible, the motion of the actual tumor, rather than the liver center of mass motion, should be used for target volume generation.

## 5 CONCLUSIONS

Liver resection/transplantation greatly affects respiratory‐induced liver motion in patients with HCC. We determined that the respiratory‐induced liver motion in HCC patients with liver resection/transplantation was smaller than that in HCC patients without liver resection/transplantation. Therefore, we recommend discriminatory ITV determination in patients with or without liver resection/transplantation undergoing external radiotherapy for hepatic tumors while respiratory motion management is unavailable.

## CONFLICT OF INTEREST

The authors declare no conflicts of interest.
